# Differential immune responses in new and old fruit fly-parasitoid associations: Implications for their management

**DOI:** 10.3389/fphys.2022.945370

**Published:** 2022-08-26

**Authors:** Rehemah Gwokyalya, Jeremy K. Herren, Christopher W. Weldon, Fathiya M. Khamis, Shepard Ndlela, Samira Abuelgasim Mohamed

**Affiliations:** ^1^ International Centre of Insect Physiology and Ecology, Nairobi, Kenya; ^2^ Department of Zoology and Entomology, University of Pretoria, Pretoria, South Africa

**Keywords:** encapsulation, melanisation, hemocytes, immunity, *Diachasmimorpha longicaudata*, *Psytallia cosyrae*, *Bactrocera dorsalis*, *Ceratitis cosyra*

## Abstract

The oriental fruit fly, *Bactrocera dorsalis* (Hendel), and marula fruit fly, *Ceratitis cosyra* (Walker), are major fruit-infesting tephritids across sub-Saharan Africa. Biological control of these pests using parasitic wasps has been widely adopted but with varying levels of success. Most studies investigating host-parasitoid models have focused on functional and evolutionary aspects leaving a knowledge gap about the physiological mechanisms underpinning the efficacy of parasitoids as biocontrol agents of tephritids. To better understand these physiological mechanisms, we investigated changes in the cellular immune responses of *C. cosyra* and *B. dorsalis* when exposed to the parasitic wasps, *Diachasmimorpha longicaudata* (Ashmaed) and *Psyttalia cosyrae* (Wilkinson). We found that *B. dorsalis* was more resistant to parasitisation, had a higher hemocyte count, and encapsulated more parasitoid eggs compared to *C. cosyra*, achieving up to 100% encapsulation when exposed to *P. cosyra*e. Exposing *B. dorsalis* to either parasitoid species induced the formation of a rare cell type, the giant multinucleated hemocyte, which was not observed in *C. cosyra*. Furthermore, compared to *P. cosyra*e-parasitized larvae, those of both host species parasitized by *D. longicaudata* had lower encapsulation rates, hemocyte counts and spreading abilities and yielded a higher number of parasitoid progeny with the highest parasitoid emergence (72.13%) recorded in *C. cosyra*. These results demonstrate that cellular immune responses are central to host-parasitoid interaction in tephritid fruit flies and further suggest that *D. longicaudata* presents greater potential as a biocontrol agent of *B. dorsalis* and *C. cosyra* in horticultural cropping systems.

## 1 Introduction

The oriental fruit fly, *Bactrocera dorsalis* (Hendel), and marula fruit fly, *Ceratitis cosyra* (Walker) (both Diptera: Tephritidae), are major fruit-infesting tephritids across sub-Saharan Africa and are responsible for more than 80% losses of cultivated fruit (Ekesi et al., 2006; Mwatawala et al., 2006). Infestation with these species presents major constraints to food security and the economies of the countries where they are prevalent (Ekesi et al., 2006; Vargas et al., 2012). Hence, devising and implementing effective control strategies for these pests has become a priority. Hymenopteran parasitoids are among the most promising biological control agents adopted in this endeavor (Ovruski et al., 2000; Mohamed et al., 2008; Ndlela et al., 2020).

*Diachasmimorpha longicaudata* (Ashmaed) (Hymenoptera: Braconidae) is a parasitoid species credited with one of the most successful biological control program undertaken against fruit flies. These programs were implemented in Hawaii (Vargas et al., 1993, 2012) and more recently in French Polynesia (Leblanc et al., 2013). *Diachasmimorpha longicaudata* has been imported and evaluated against native Ceratitis species and the invasive fruit fly *B. dorsalis* (Mohamed et al., 2008; Ndlela et al., 2020). Currently this parasitoid has been released in over 10 African countries with encouraging results in suppression of the invasive fruit fly, *B. dorsalis* (Mohamed et al., 2016). On the other hand, *Psyttalia cosyrae* (Wilkinson) (Hymenoptera: Braconidae) has been proven to be a very efficient parasitoid against its co-evolved host *C. cosyra* (Mohamed et al., 2008; Ndlela et al., 2020), hence it represents an ideal candidate for augmentative release for biological control of this pest and other related species.

Studies of parasitoids’ functional response have demonstrated that host preference and parasitism success as well as host fly susceptibility to parasitism vary across several host-parasitoid associations in tephritid fruit flies (Mohamed et al., 2003, 2008; Wang and Messing, 2004; Segura et al., 2012; Sá et al., 2018; Harbi et al., 2019; Ndlela et al., 2020). Parasitoid species performing well in one host may achieve very low or no parasitism in other hosts. For instance, Ndlela et al. (2020) reported that *D. longicaudata* achieved higher parasitism rates when exposed to both *B. dorsalis* and *C. cosyra* compared to when these tephritids were parasitized by *P. cosyra*e. Likewise, *P. cosyra*e successfully parasitized *C. cosyra*, but no parasitoid emergence occurred when it was exposed to *B. dorsalis*. Moreover, Mohamed et al. (2003) reported that *P. cosyra*e yielded higher parasitism when exposed to both *C. cosyra* and *C. capitata* but none when exposed to other *Ceratitis* species and *Zeugodacus* (=*Bactrocera*) *cucurbitae* (Coquillett) (Diptera: Tephritidae) in which the eggs of this koinobiont parasitoid were encapsulated. In a later study, *D. longicaudata* was found to perform best on *C. capitata* and *C. cosyra*, followed by *B. dorsalis* while it failed to yield viable parasitoid progeny in *Ceratitis fasciventris* (Bezzi) and *Ceratitis anonae* Graham (Mohamed et al., 2008). The same authors reported that the eggs of this parasitoid were encapsulated in *B. dorsalis* and the latter two *Ceratitis* species, hence the low parasitism in these hosts. While these studies provide insights into the ecological dynamics of host-parasitoid systems of tephritid fruit flies, the fundamental physiological mechanisms underpinning these interactions are poorly understood and often disregarded, which jeopardizes effective management of these pests.

For successful parasitism to occur, the parasitoid offspring must circumvent host immune defenses by actively suppressing host immunity or employing passive approaches like molecular mimicry and avoidance of immune detection (Monconduit and Prevost, 1994; Eslin and Prévost, 2000; Nappi et al., 2009). There is evidence of successful host immune evasion by certain parasitoids while others only partially survive, and in some instances the parasitoid offspring completely fail to develop within the host (Mohamed et al., 2003, 2008; Kacsoh and Schlenke, 2012; Ndlela et al., 2020). A few studies have linked high parasitism success to parasitoid factors that downregulate host immunity whereas others accrued the same success as a consequence of weak host immune mechanisms that cannot mount defense responses to overcome parasitism (Eslin and Doury, 2006).

Upon attack by parasitic wasps, insects deploy both humoral and cellular mechanisms, which work in tandem (Schmid-Hempel, 2009) and mount defense mechanisms like encapsulation and melanisation. If the immune responses are successful the host survives, otherwise, the parasitoid survives and kills the host insect. Encapsulation involves the aggregation of host hemocytes around the parasitoid egg to form a capsule (Schmidt et al., 2001; Strand, 2008; Kacsoh and Schlenke, 2012). Capsule formation is followed by hemocyte lysis and melanisation of the capsule-forming hemocytes, eventually killing the parasitoid offspring via asphyxiation and/or production of reactive oxygen species (Schmidt et al., 2001; Dudzic et al., 2015).

The ability of a host to successfully encapsulate invading parasitic wasps is largely dependent on its circulating hemocyte numbers. This hypothesis is supported by observations that insects with higher hemocyte counts exhibit higher encapsulation rates making them more resistant to parasitic wasps (Eslin and Prevost, 1996; Eslin and Prévost, 1998; Moreau et al., 2005; Kacsoh and Schlenke, 2012). In addition to the higher total hemocyte counts (THCs), the presence of certain hemocyte types is crucial for mounting effective defense responses. In some *Drosophila* species, for instance, inhibition of lamellocyte and crystal cell production impairs encapsulation and melanisation responses, respectively, in infected hosts (Binggeli et al., 2014; Trainor et al., 2021). The same has been demonstrated in some Lepidopterans when plasmatocyte and granulocyte proliferation and activity were suppressed (Pech and Strand, 1996; Chiu and Govind, 2002; Cho and Cho, 2019). Coupled with the above mechanisms, interfering with hemocyte spreading and adhesion (Rizki and Rizki, 1994; Cho and Cho, 2019) reduces the hosts’ encapsulation ability, making them more susceptible to parasitisation.

These observations indicate that cellular-mediated immune responses are key drivers of host-parasitoid models and that they can be used as markers to better understand the dynamics of host-parasitoid interactions. Very few studies have attempted to explore the immune mechanisms underlying the diverse host-parasitoid phenotypes in tephritid fruit flies with the majority only speculating about the possible mechanisms involved in these host-parasitoid systems. Yet, to improve biological control of these horticultural pests using parasitoids, it is prudent that a physiological model of these hosts’ cellular-mediated immune mechanisms is investigated to facilitate informed decision making for improved management of these pests, especially in species-specific systems.

Hence, in this study we investigated whether melanotic encapsulation responses of *B. dorsalis* and *C. cosyra* varied when these tephritid fruit flies are exposed to *D. longicaudata* and *P. cosyra*e. We then proceeded to investigate whether these variations in melanotic encapsulation were influenced by host intrinsic factors such as hemocyte load, constitutive hemocyte types and the spreading ability of the host hemocytes. We predicted that the host species with the highest probability to encapsulate parasitoid eggs would have higher circulating THCs and it would eventually be more resistant to parasitisation.

## 2 Material and methods

### 2.1 Host and parasitoid culturing and maintenance

Insect cultures of *C. cosyra* and *B. dorsalis* used in this study were collected from Machakos county (01°14′S, 37°23′E), Kenya, and reared in the insectary at the International Centre of Insect Physiology and Ecology (icipe) in Perspex cages (80 cm × 80 cm × 80 cm) at 25–27°C, 60%–70% relative humidity, and 12:12 days: light photoperiod. Ripe mangoes were exposed to the adults for oviposition, after which the eggs were allowed to hatch, and the emerging larvae fed on and developed in the mango. Fresh mango puree was supplemented *ad libitum* depending on the larval population. Before pupation, third instar larvae were transferred to a petri dish containing sterile sand to allow burrowing and subsequent pupation. Post-eclosion, the adults were fed on a mixture of sugar and enzymatic yeast hydrolysate (Ndlela et al., 2020) and water. The parasitoids used in this study were reared together with fruit flies under similar conditions as described above. *Psyttalia cosyrae* was reared on *C. cosyra* second instar larvae as hosts while *D. longicaudata* was raised on *B. dorsalis* second instar larvae as described by Mohamed et al. (2008). Eclosed parasitoid adults were fed on a 50% (v/v) honey solution *ad libitum*.

### 2.2 Exposure to parasitoids

To obtain suitable larval stage specimen for parasitisation assays, pesticide-free ripe mangoes were exposed to adult fruit flies (>5 days old) for oviposition and the eggs were left to hatch. The emerging larvae were reared as described above till they reached the second instar at which they were used for bioassays. One hundred-second instar of each of *B. dorsalis* and *C. cosyra* host larvae were randomly selected and separately transferred to parasitoid oviposition units (Ndlela et al., 2020). The oviposition units were transferred to Perspex cages (12 cm × 12 cm × 12 cm) containing 4-day old, 10 naive female parasitoid wasps. Prior to the bioassays, we observed that using naïve parasitoids at a 1:10 parasitoid: host ratio, *D. longicaudata* achieved ≥90% oviposition when host larvae exposure was done for 2 hours and *P. cosyra*e achieved the same oviposition rate when exposure was done for 6 hours (data not reported here). As such, for all experiments, exposure to *D. longicaudata* was done for 2 hours while exposure to *P. cosyra*e was done for 6 hours. After exposure to parasitoids, the host larvae were transferred to fresh carrot diets (Mohamed et al., 2003) and incubated at 27°C, 60%–70% humidity, and 12:12 days: light photoperiod till subsequent bioassays. All bioassays were conducted 24- and 36 h post parasitisation (hpp) unless stated otherwise.

### 2.3 Encapsulation and melanisation assays

To investigate the encapsulation and melanisation abilities of both species when parasitized, host larvae were retrieved from the carrot diet and briefly washed in sterile water. Each larva was dissected in phosphate buffered saline (PBS) under a stereomicroscope (ZEISS Stemi 508, ZEISS, Oberkochen, Germany) and checked for the parasitoid immature stages (a layer of hemocytes surrounding the parasitoid egg or larva) and melanisation (black/brown hemocyte layers around the parasitoid egg) status. For each experimental group (*B. dorsalis* exposed to *D. longicaudata*, *B. dorsalis* exposed to *P. cosyra*e, *C. cosyra* exposed to *D. longicaudata*, *C. cosyra* exposed to *P. cosyra*e), a total of 50 parasitized larvae were dissected and replicated four times making the total dissected larvae of each group 200.

### 2.4 Hemolymph collection

Host larvae that had been offered to each parasitoid species were removed from the carrot diet, washed in PBS, sterilized using 70% ethanol, and anaesthetized on ice. Hemolymph was collected by piercing the larval cuticle at the fourth segment with a sterile needle from only those larvae that were parasitized. To confirm parasitisation, each larva was dissected after hemolymph collection; if a larva had parasitoid offspring (egg or larva), its hemolymph was used for subsequent bioassays. The hemolymph of unparasitized larvae was discarded. Unexposed host fruit fly larvae were used as controls.

### 2.5 Total hemocyte counts

Total hemocyte count (THC) assays were conducted according to Altuntaş et al. (2012) with slight modifications. Briefly, 2 μl of hemolymph were collected from each parasitized larva as described in Section 2.4 using a micropipette and immediately transferred to a 1.5 ml microcentrifuge tube containing 18 μl of ice-cold anti-coagulant buffer (0.098 M NaOH, 0.186 M NaCl, 0.017 M Na2EDTA and 0.041 M citric acid, pH 4.5). From the mixture, 10 μl were aliquoted, loaded on each side of an improved Neubauer hemocytometer, and the hemocytes were counted under a light microscope (Leica DM 2500 LED, Leica microsystems, Wetzlar, Germany). Total hemocyte counts were expressed as the number of hemocytes × 10^5^/ml hemolymph. The hemolymph was individually collected from 30 larvae for each experimental group, and this was replicated four times. Unexposed fruit fly larvae were used as a control (30 individuals in four replicates).

### 2.6 Differential hemocyte counts

Differential hemocyte counts (DHC) were conducted using a modified protocol as that described by Altuntaş et al. (2012). Hemolymph was collected as described in Section 2.4 and the host larvae were bled on pre-cleaned microscope slides. The hemolymph was spread on the microscope slide and left to dry at room temperature after which the hemocytes were fixed using a methanol: glaciated acetic acid (3:1) solution. Post-fixation, the hemocytes were stained with 5% Giemsa/PBS (v/v) solution for 30 min and the slides were rinsed with distilled water. Hemocyte visualization was done using a light microscope (Leica DM 2500 LED, Leica microsystems, Wetzlar, Germany) and classification was done according to Gupta, (1979). Five groups of hemocytes (prohemocytes, plasmatocytes, granulocytes, spherulocytes and oenocytes) were identified in *C. cosyra* while six hemocyte types (prohemocytes, plasmatocytes, granulocytes, spherulocytes, oenocytes, and adipohemocytes) were identified in *B. dorsalis*. In addition to characterizing the hemocyte types of the host larvae, we investigated the changes in the different hemocytes of both host species post-parasitisation. For this, a total of 500 cells were randomly counted from each slide, the different hemocyte types were recorded and expressed as percentages. The hemolymph was individually collected from 15 larvae for each experimental group, and this was replicated four times.

The hemocyte images were corrected for exposure, contrast and brightness using the Leica LASZ software and the resolution adjusted using Adobe Illustrator CC 2020 (version 24.2).

### 2.7 Hemocyte spreading assays

To gain insight into the possible mechanisms with which parasitoids suppress host melanotic encapsulation responses, a cell spreading assay was conducted 36hpp using a modified procedure as that described by Gwokyalya and Altuntaş, (2019). Briefly, 2 μl of hemolymph were collected as described in Section 2.4 and mixed with 10 μl of chilled PBS in a 1.5 ml microcentrifuge tube. Ten microliters of the mixture were spread on a pre-cleaned microscope slide and incubated in a humidifying water bath at 30°C for 30 min. Post-incubation, the slides were rinsed with distilled water and the hemocytes were visualized under a light microscope (Leica DM 2500 LED, Leica microsystems, Wetzlar, Germany). From each slide, a total of 100 cells were examined and the number of spread cells were counted, and their proportion was expressed as a percentage.

### 2.8 Cell viability assay

The viability of the host hemocytes was investigated 36 hpp using the trypan blue exclusion method described by Coates et al. (2019) with modifications. Briefly, 2 µl of hemolymph were extracted from each host larva as described in Section 2.4, transferred to a 1.5 ml microcentrifuge tube containing 6 µl of ice-cold 0.02% trypan blue-PBS solution, and incubated for 3 min. The hemocyte-trypan blue mixture was loaded on a Neubauer hemocytometer, and the hemocytes visualized under a light microscope (Leica DM 2500 LED, Leica microsystems, Wetzlar, Germany). The live (unstained) and dead (blue-stained) hemocytes were counted, recorded, and expressed as a percentage of the total number of hemocytes counted. The bioassay was done using 15 larvae for each experimental group, and this was repeated four times.

### 2.9 Parasitoid emergence assay

To check for variations in parasitoid emergence for the different host-parasitoid associations (*B. dorsalis* to *D. longicaudata*, *B. dorsalis* to *P. cosyra*e, *C. cosyra* to *D. longicaudata*, and *C. cosyra* to *P. cosyra*e) exposure of host larvae to parasitoids was performed as described in Section 2.2. The parasitized larvae were retrieved from the carrot diets and transferred to fresh carrot diets till pupation. The emerging pupae were then monitored daily for fly and/or parasitoid emergence. These assays were replicated four times for each treatment group. The number of emerging parasitoids was expressed as a proportion of the total number of host pupae to reflect parasitism success.

### 2.10 Data analysis

All data analyses were performed using R software (version 4.0.1) (R Core Team, 2021). Encapsulation and melanisation data were analyzed using a generalized linear model (GLM) assuming a binomial distribution error with parasitoid (two species), host (two fruit fly species) and time (two levels) as factor effects.

For each host fruit fly species, THCs and DHCs were evaluated for each parasitized group at 24- and 36-h after exposure to parasitoids and these were compared with the host THCs and DHCs recorded in the unexposed control groups at the start of the experiment (0 h). As such, the unexposed larval groups and each parasitized group of each host fruit fly at the different time points (*D. longicaudata* and *P. cosyra*e at 24 and 36 h, each) was considered as a treatment group (five treatment groups in total) during analysis. A GLM assuming a Poisson distribution error was used to analyze the effect of exposure to parasitoids on the THCs of each fruit fly species. Differential hemocyte counts (plasmatocyte, granulocyte, spherulocyte, oenocyte, prohemocyte and adipohemocyte) of each host species were analyzed using beta regression that assumes the beta binomial distribution error with betareg package (Cribari-Neto and Zeileis, 2010) considering the treatment groups (Control, *D. longicaudata* at 24 h, *D. longicaudata* at 36 h, *P. cosyra*e at 24 h, and *P. cosyra*e at 36 h) as factors. When recording the differential hemocytes, we observed the presence of giant multinucleated hemocytes (GMHs) in *B. dorsalis*; these were recorded and analyzed using a GLM with Poisson error distribution with time after parasitisation and parasitoid species as fixed factors.

Proportion data on hemocyte viability and spreading were analyzed using beta regression that assumes the beta binomial distribution error with betareg package (Cribari-Neto and Zeileis, 2010) considering host and parasitoid species as fixed factors in the model. To compare the effect of host and parasitoid species on parasitism success, a GLM assuming a quasibinomial distribution error was performed.

The significance of the models was determined using analysis of deviance with chi-square tests. Mean separation was done using Tukey’s multiple comparison tests at *p* ≤ 0.05, using the “lsmeans” package. To investigate whether the changes in the recorded immune changes were correlated with the parasitoid emergence rates, a Pearson correlation coefficient analysis was conducted separately, for each host fruit fly.

## 3 Results

### 3.1 Host encapsulation and melanisation responses

**FIGURE 1:**
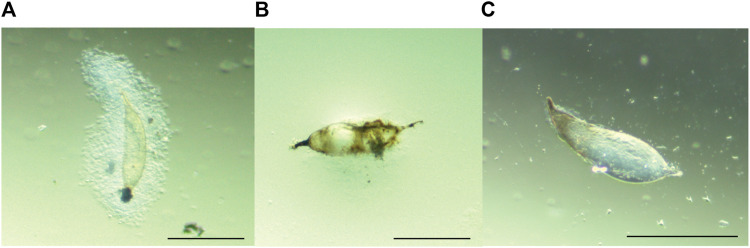
Melanotic encapsulation responses of **(A,B)** *B. dorsalis* and **(C)** *C. cosyra*. Figure 1A was taken at 24 hpp, Figures 1B,C were taken 36 hpp. Scale bars = 300 μm.

*Ceratitis cosyra* and *B. dorsalis* variably encapsulated the eggs of *D. longicaudata* and *P. cosyra*e. Encapsulation responses in *B. dorsalis* were characterized by large capsules consisting of several thick layers of hemocytes surrounding the parasitoid egg. These capsules were accompanied by melanisation of the hemocyte layers and eventually the parasitoid egg itself (Figures 1A,B). For *C. cosyra*, a very thin layer of hemocytes was observed surrounding the parasitoid egg, which was occasionally accompanied by partial melanisation of the parasitoid egg (Figure 1C).

**FIGURE 2:**
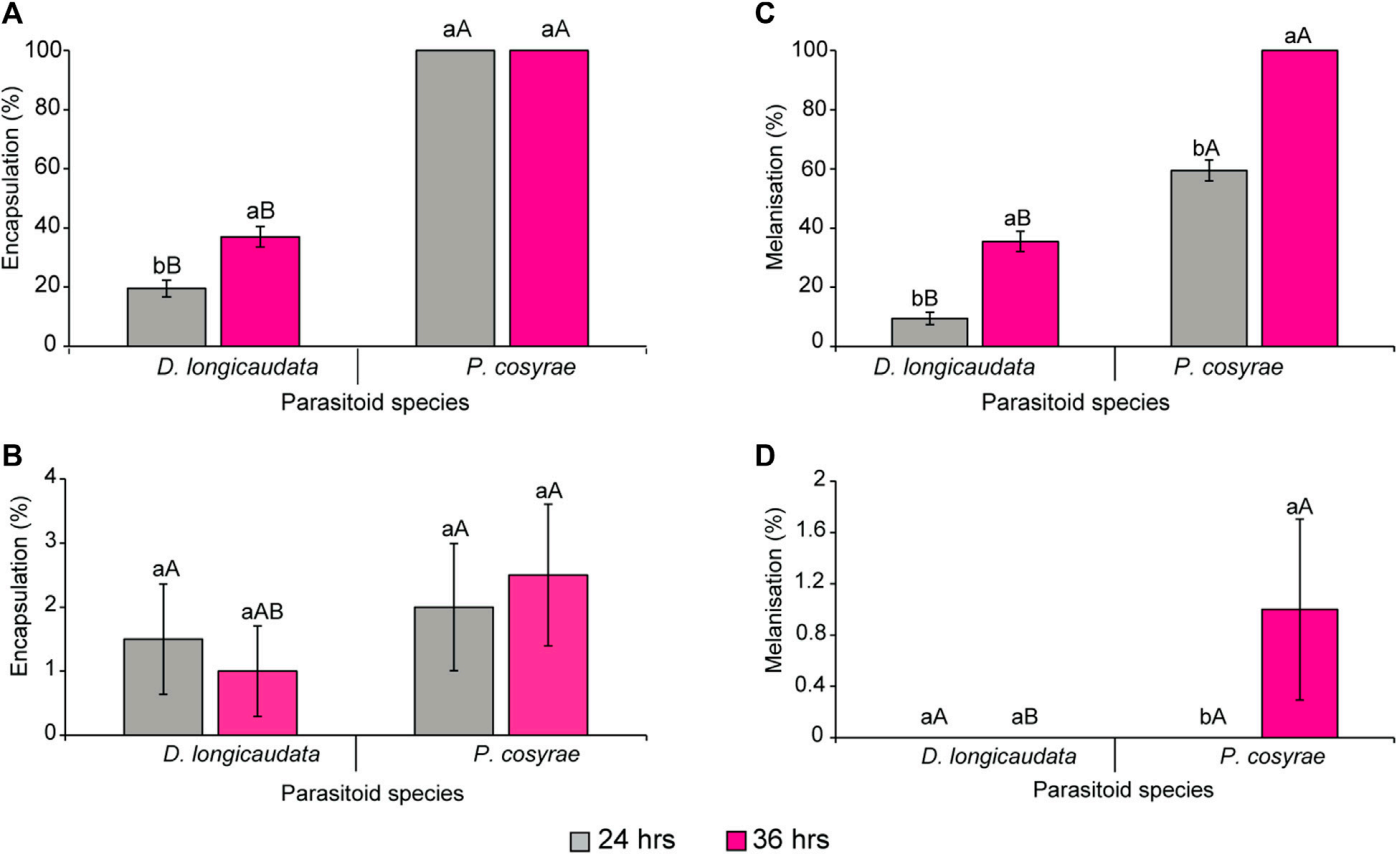
Encapsulation rates of **(A)** *B. dorsalis* and **(B)** *C. cosyra*, and melanisation rates of **(C)** *B. dorsalis* and **(D)** *C. cosyra*. Within the same parasitoid species group exposed to each host fruit fly, bars capped with different letters (lowercase letters denote differences within the different time points of the same parasitoid group while upper case letters denote differences between the different parasitoids analyzed at the same time point) are statistically different (Tukey tests: *p* ≤ 0.05, *n* = 200). Error bars indicate ± standard error of the mean (SEM).

*Ceratitis cosyra* and *B. dorsalis* differentially encapsulated the eggs of *D. longicaudata* and *P. cosyra*e as evidenced by the significant effect of fly species (LR χ^2^ = 1,119.91, df = 1, *p* < 0.0001), parasitoid (LR χ^2^ = 532.21, df = 1, *p* < 0.0001), time (LR χ^2^ = 13.06, df = 1, *p* = 0.003) and of the interaction effect of fruit fly species × parasitoid species (LR χ^2^ = 45.81, df = 1, *p* < 0.0001). Overall, *B. dorsalis* encapsulated more parasitoid eggs compared to *C. cosyra* regardless of parasitoid species. Notably, *B. dorsalis* achieved up to 100% encapsulation rates when subjected to parasitism by *P. cosyra*e as early as 24 hpp (Figure 2A), while for *C. cosyra*, encapsulation was 2% for the same parasitoid species at the same time point. (Figure 2B). Comparing the two parasitoid species on the same host species, no significant differences were recorded in *C. cosyra*’s encapsulation rates for both parasitoids, whereas in *B. dorsalis*, encapsulation was lower for *D. longicaudata* eggs compared to *P. cosyra*e’s at both time points. At 36 hpp, all *P. cosyra*e eggs in *B. dorsalis* were melanized hence no encapsulation was recorded (Figures 2A,C).

While there was significant three-way interaction effect of the factors under study with encapsulation response, there was no significant interaction between fruit fly species, parasitoid species, and time, neither were the two-way interaction effect of the factors since the melanisation responses recorded for *C. cosyra* on each parasitoid species at each time point were almost negligible. However, the main effect of fruit fly species (LR χ^2^ = 890.23, df = 1, *p* < 0.001), parasitoid species (LR χ^2^ = 337.69, df = 1, *p* < 0.001) and time after parasitisation (LR χ^2^ = 148.68, df = 1, *p* < 0.001) were significant. Up to 100% melanisation was observed in *B. dorsalis* exposed to *P. cosyra*e at the 36 h time point, whereas for *D. longicaudata*, melanisation was 35.5% at the 36 h time point (Figure 2C). While we recorded a 1% melanisation rate for *P. cosyra*e exposed to *C. cosyra*, none of the *D. longicaudata* eggs was melanized in *C. cosyra* at both time points (Figure 2D).

### 3.2 Effects of parasitisation on host total hemocyte counts

**FIGURE 3:**
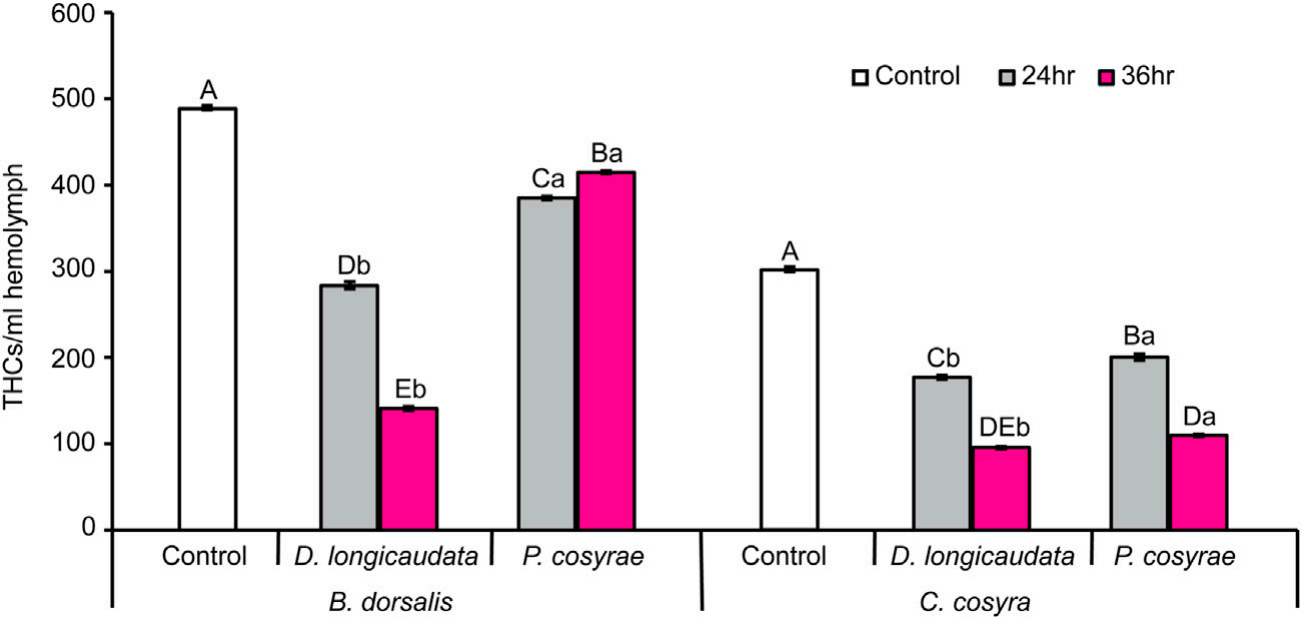
Mean host total hemocyte counts over time in *B. dorsalis* and *C. cosyra* in response to exposure to the parasitic wasps, *D. longicaudata* and *P. cosyra*e. Bars capped with different letters within each host species (lowercase letters denote differences across all treatment groups of the same host species while upper case letters denote differences between the different parasitoids analysed at the same time point) are statistically different (Tukey tests: *p* ≤ 0.05, *n* = 120). Error bars indicate ± SEM.

Total hemocyte counts were highly variable in each host species across all treatment groups. Overall, *B. dorsalis* had more hemocytes/ml hemolymph with up to twice as many total hemocytes as *C. cosyra* (Figure 3). However, these numbers plummeted post-parasitisation: the THCs in parasitized *B. dorsalis* larvae were significantly lower than that of their control counterparts (LR χ^2^ = 847.93, df = 4, *p* < 0.001) with the largest reduction in THCs found in the *D. longicaudata*-parasitized larval group analyzed at 36 hpp (71.18% relative to the control). However, while *D. longicaudata* consistently reduced the THCs of *B. dorsalis*, the same trend was not recorded for *P. cosyra*e-parasitized larval groups where THCs were higher at 36 hpp compared to those recorded at the 24 h time point (Figure 3).

In *C. cosyra*, THCs significantly varied with exposure to parasitic wasps (LR χ^2^ = 596.24, df = 4, *p* < 0.001) compared with their control counterparts. Both *D. longicaudata* and *P. cosyra*e consistently reduced the THCs of *C. cosyra* but the lowest THCs (68.19% reduction relative to the control) were recorded in the *D. longicaudata*-parasitized larval groups analyzed at 36 hpp (Figure 3).

### 3.3 Effects of parasitisation on host differential hemocyte counts

**FIGURE 4:**
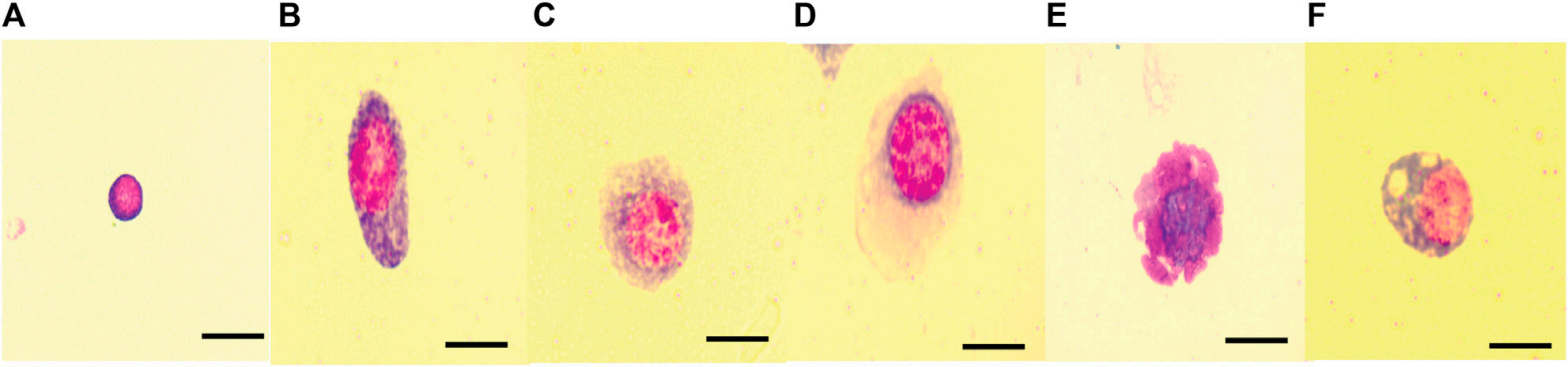
Hemocyte types of *B. dorsalis* and *C. cosyra*. **(A)** Prohemocyte, **(B)** plasmatocyte, **(C)** granulocyte, **(D)** oenocyte, **(E)** spherulocyte, **(F)** adipohemocyte (only in *B. dorsalis*). (Prohemocyte, plasmatocyte and adipocyte images were taken from *B. dorsalis* larvae microscopic slides; Granulocyte, oenocyte and spherulocyte images were taken from *C. cosyra* larvae microscopic slides. Scale bars = 10 μm.

Hemocyte classification revealed five different hemocyte types (plasmatocytes, granulocytes, prohemocytes, spherulocytes, and oenocytes) in *C. cosyra* while six hemocyte types (plasmatocytes, granulocytes, spherulocytes, oenocytes, prohemocytes and adipocytes) were recorded in *B. dorsalis*. Prohemocytes appeared as small (7–10 μm in diameter) round or oval cells containing a purple or pink stained centrally placed nucleus with a high nucleus: cytoplasm ratio (Figure 4A).

Plasmatocytes, which measured 13–42 μm, varied in morphology. In general, they appeared as round, oval, fusiform, spindle shaped, or spherical cells with centrally located circular or ovoid nuclei. Their cytoplasm contained fine granules and vacuoles. The plasma membrane was non-uniform with several vesicular invaginations (Figure 4B).

Granulocytes were also observed in larvae of both host species. These were polymorphic with centrally placed purple-reddish stained nuclei. Their cytoplasm appeared granular and contained several vacuoles. Granulocytes measured 10–45 µm in diameter (Figure 4C). Plasmatocyte-granulocyte intermediates were also observed indicating differentiation of plasmatocytes into granulocytes.

Oenocytes appeared as ovoid-shaped cells of 15–40 μm diameter. They contained a smooth homogenous cytoplasm with an eccentrically located nucleus (Figure 4D). Spherulocytes (13–27 μm in diameter) appeared as irregularly shaped cells with centrally place nuclei and cytoplasm containing prominent spherules and/or granules which almost obscured the nuclei. The plasma membrane was characterized by pronounced vesicular invaginations (Figure 4E).

Adipocytes, which were recorded only in *B. dorsalis*, appeared as round or oval-shaped cells of 15–25 μm diameter whose granular cytoplasm contained several vacuoles and fat droplets. Their nuclei appeared circular and were centrally placed (Figure 4F).

In both host species, plasmatocytes were the most dominant hemocyte type, constituting 81–95% of the THCs followed by granulocytes (13%). The other cells were present at very low numbers, constituting 2–4% of the THC (Table 1).

**TABLE 1:** Percentage of the different hemocyte types of *C. cosyra* and *B. dorsalis* before and after exposure to *D. longicaudata* and *P. cosyrae*. Percentages are based on random counts of 500 cells.

Host species	Hemocyte type (%)	Control	24 h		36 h	
			*D. longicaudata*	*P. cosyrae*	*D. longicaudata*	*P. cosyrae*
*B. dorsalis*	Plasmatocytes	92.36 ± 0.125a	82.30 ± 0.242bB	83.36 ± 0.256bA	94.59 ± 0.122aA	81.42 ± 0.153cB
	Granulocytes	5.50 ± 0.109d	13.70 ± 0.251aA	12.28 ± 0.225bA	3.93 ± 0.110eB	11.07 ± 0.145cA
	Prohemocytes	1.61 ± 0.041a	1.59 ± 0.058aA	1.70 ± 0.061aA	1.22 ± 0.045cB	1.45 ± 0.044bB
	Oenocytes	0.29 ± 0.025d	1.85 ± 0.066bA	2.19 ± 0.063cB	0.14 ± 0.019eB	5.45 ± 0.078aA
	Spherulocytes	0.06 ± 0.014b	0.07 ± 0.015bB	0.14 ± 0.021aA	0.13 ± 0.019aA	0.09 ± 0.015abB
	Adipohemocytes	0.18 ± 0.021c	0.49 ± 0.043aA	0.33 ± 0.024bB	0.0 ± 0.000dB	0.54 ± 0.029aA
*C. cosyra*	Plasmatocytes	94.59 ± 0.122a	92.48 ± 0.129bA	91.35 ± 0.156 abA	91.03 ± 0.140abB	88.37 ± 0.166cB
	Granulocytes	3.89 ± 0.116c	5.38 ± 0.123aA	4.89 ± 0.131 abB	5.26 ± 0.126aA	5.52 ± 0.123aA
	Prohemocytes	1.23 ± 0.047d	0.98 ± 0.033eB	1.74 ± 0.052 bB	1.30 ± 0.039cA	2.06 ± 0.044aA
	Oenocytes	0.15 ± 0.019e	1.00 ± 0.032dB	1.83 ± 0.053cB	2.18 ± 0.046bA	3.85 ± 0.108aA
	Spherulocytes	0.15 ± 0.023c	0.17 ± 0.018bcB	0.20 ± 0.025 bA	0.23 ± 0.020aA	0.20 ± 0.019bA

Values are presented as percent mean ± SEM. For each hemocyte type, means with different letters across treatment groups (lowercase letters denote differences across all treatment groups while upper case letters denote differences between time points for the same parasitoid group) are significantly different. (*p* ≤ 0.05, *n* = 60).

Analysis of the DHCs of *B. dorsalis* host larvae showed a significant effect of parasitoid exposure on the larval plasmatocyte counts (LR χ^2^ = 2,888.30, df = 4, *p* < 0.001), plasmatocyte counts were lower across all parasitized larval groups except for the *D. longicaudata*-parasitized larvae analyzed at 36 hpp whose plasmatocyte counts did not differ from the control (Table 1). Likewise, prohemocyte counts significantly reduced after exposure to parasitoids (LR χ^2^ = 71.89, df = 4, *p* < 0.001) with the least prohemocyte counts (1.2 ± 0.04%) recorded in *D. longicaudata*-parasitized larvae analyzed at 36 hpp. In contrast, we noted that exposure to parasitoids had a significant positive effect on the granulocyte counts of *B. dorsalis* (LR χ^2^ = 1,955, df = 4, *p* = 0.006) except in *D. longicaudata*-parasitized larvae evaluated at 36 hpp where granulocyte numbers reduced by 20% compared to the control. Similarly, oenocytes significantly increased post-parasitisation (LR χ^2^ = 2,228.90, df = 4, *p* < 0.001) especially at 36 hpp in *P. cosyra*e-parasitized larvae where we recorded a 27-fold increase in oenocyte numbers relative to the control. Additionally, exposing *B. dorsalis* to parasitoids significantly influenced adipohemocyte counts (LR χ^2^ = 1,955, df = 4, *p* < 0.001). *Bactrocera dorsalis* larvae exposed to *D. longicaudata* and analyzed at 24 hpp had the highest adipohemocyte counts. We however did not record any adipohemocytes for the same parasitoid-exposed group at 36 hpp. Spherulocytes were also significantly affected by exposure to parasitic wasps (LR χ^2^ = 27.78, df = 4, *p* < 0.001) (Table 1).

In *C. cosyra*, we found that exposing host larvae to parasitoids significantly affected the proportion of plasmatocytes (LR χ^2^ = 1,053.40, df = 4, *p* < 0.001) with the lowest plasmatocyte counts (88.37%) recorded in larval groups exposed to *P. cosyra*e and analyzed at 36 hpp. In contrast, granulocyte counts of *C. cosyra* significantly increased due to parasitoid exposure (LR χ^2^ = 147.64, df = 4, *p* < 0.001), the highest proportion of granulocytes were recorded in *P. cosyra*e-parasitized larval groups examined at 36hpp. Similar findings were recorded for oenocyte (LR χ^2^ = 844.81, df = 4, *p* < 0.001) and prohemocyte counts (LR χ^2^ = 315.70, df = 4, *p* < 0.001) except that at 24 hpp prohemocyte counts were lower in *C. cosyra* larvae exposed to *D. longicaudata*, compared to the control counterparts (Table 1). Spherulocyte counts of *C. cosyra* were also significantly influenced by exposure to parasitic wasps (LR χ^2^ = 15.04, df = 4, *p* < 0.001) (Table 1).

### 3.4 Formation of giant multinucleated hemocytes in *B. dorsalis*

**FIGURE 5:**
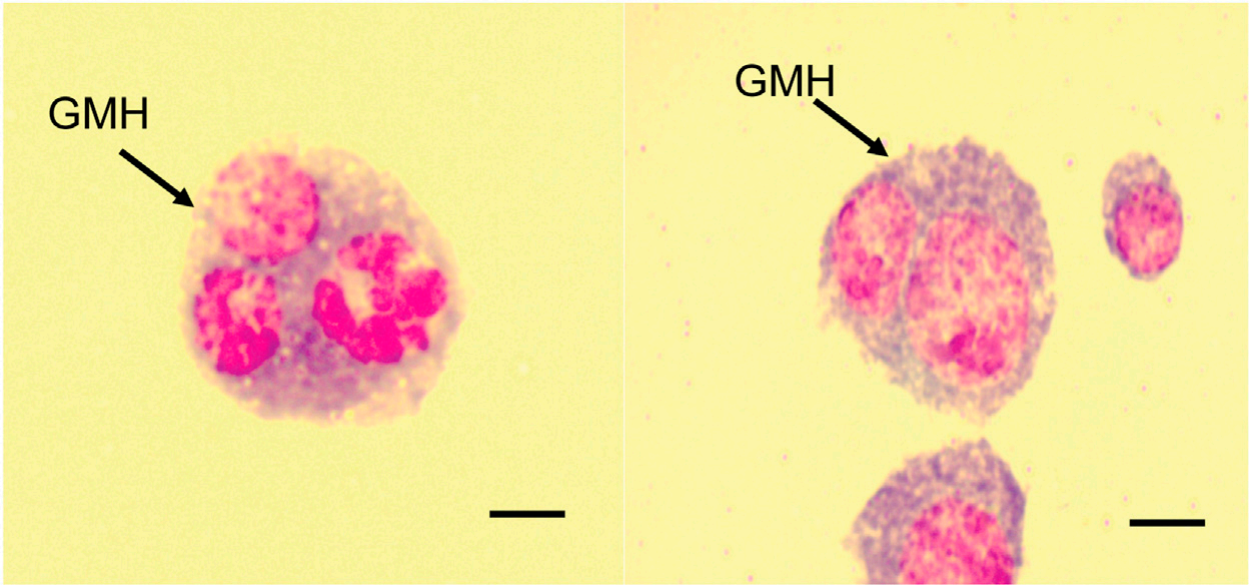
Giant multinucleated hemocytes of parasitized *B. dorsalis* host larvae. Scale bar = 10 μm.

**FIGURE 6:**
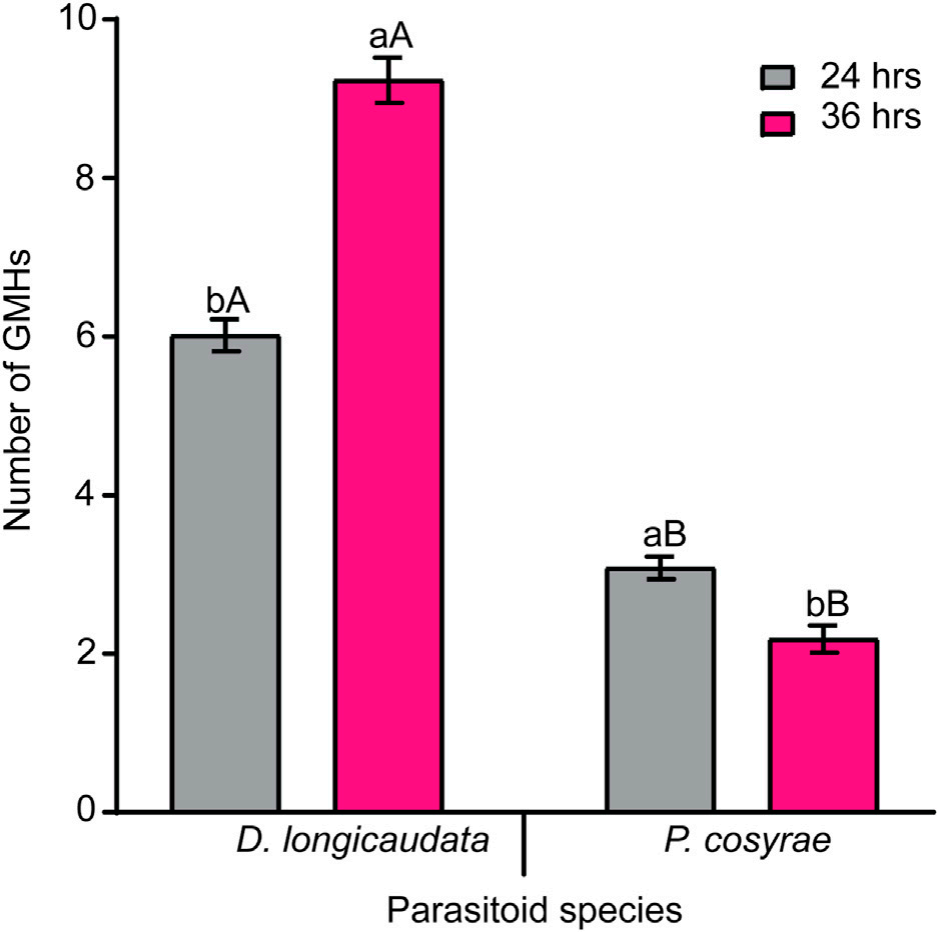
Parasitic wasp-induced formation of GMH in *B. dorsalis*. Bars capped with different letters (lowercase letters denote differences within time points of the same parasitized groups while upper case letters denote differences between the different parasitoids analyzed at the same time point) are statistically different (Tukey tests: *p* ≤ 0.05, *n* = 60). Error bars indicate ± SEM.

In addition to the different hemocytes recorded above, we noted the presence of another hemocyte type, the giant multinucleated hemocytes (GMH) (Figure 5). These hemocytes appeared as large (30–50 µm diameter) circular cells containing 2–4 nuclei. Unlike the normal mitotic cells which exhibited cytokinesis and clear spindle formation, we did not observe any occurrence of cytokinesis nor spindle formation in the GMHs. Giant multinucleated hemocytes were recorded in parasitized *B. dorsalis* larvae but not in the unexposed *B. dorsalis* control larvae nor *C. cosyra* irrespective of immune challenge. Analysis of GMH counts in *B. dorsalis* showed a significant effect of parasitoid species (LR χ^2^ = 21.75, df = 1, *p* < 0.001) on GMH formation with *D. longicaudata*-parasitized larval groups forming more GMHs compared to those parasitized by *P. cosyrae* (Figure 6). However, we recorded no significant effect of time (LR χ^2^ = 1.50, df = 1, *p* = 0.221) nor the interaction between time and parasitoid species (LR χ^2^ = 3.03, df = 1, *p* = 0.082) on GMH formation.

### 3.5 Nuclear aggregation in host hemocytes

**FIGURE 7:**
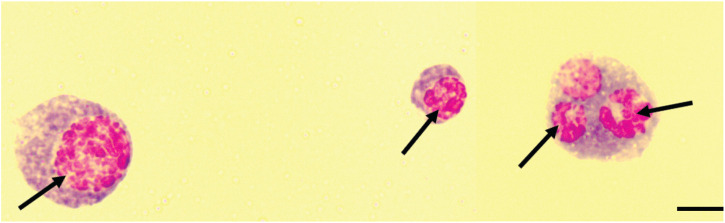
Karyorrhexis-like aggregation of the nuclei of host hemocytes. Black arrows indicate fragmented nuclei, Scale bars = 10 μm.

We further noted that exposing *B. dorsalis* and *C. cosyra* to *D. longicaudata* led to the formation of multiple polymorphic aggregates in the nuclei of the host hemocytes. These aggregates were reminiscent of karyorrhexis apoptotic cells indicating the induction of apoptotic hemocyte death by *D. longicaudata* (Figure 7).

### 3.6 Effects of parasitisation on host hemocyte spreading

**FIGURE 8:**
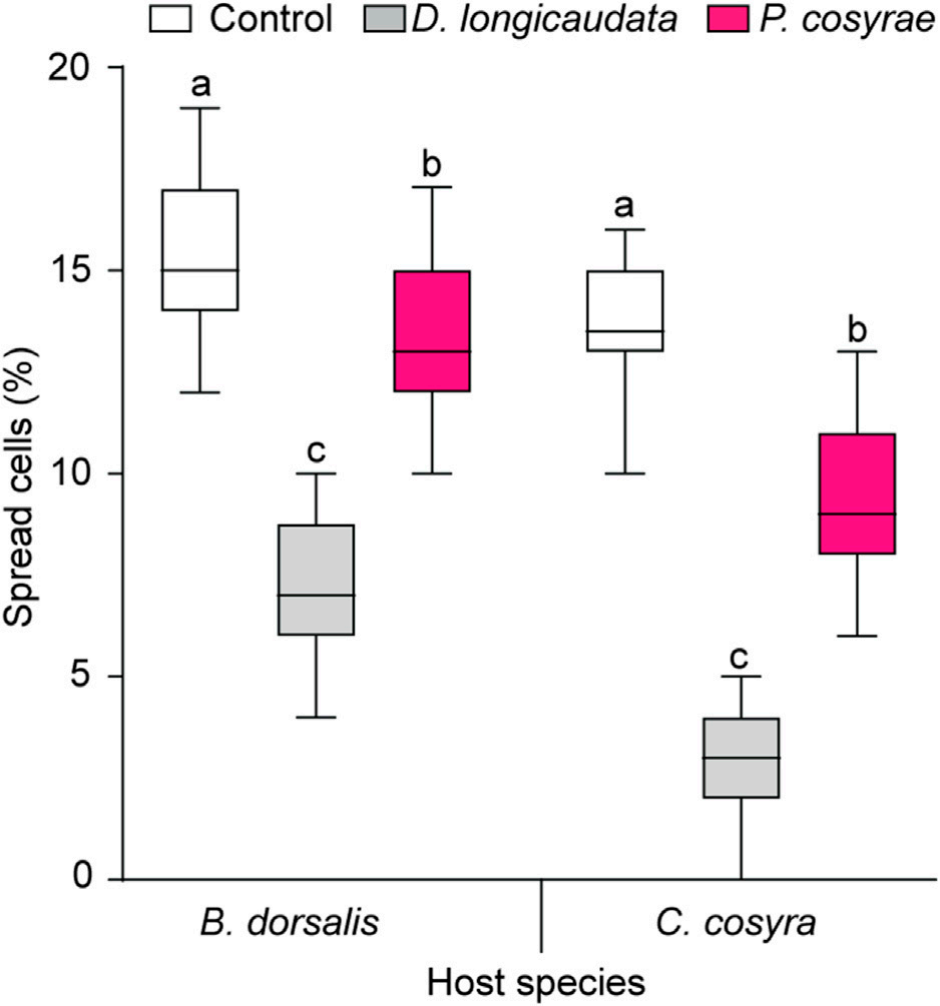
Host hemocyte spreading ability 36 h after exposure to *D. longicaudata* and *P. cosyra*e. Within each host fruit fly species, boxes with different lower-case letters are statistically different (Tukey tests: *p* ≤ 0.05, *n* = 60). Within each box, horizontal lines denote the median values and the ends of each boxplot whisker represent the minimum and maximum values of the data.

The ability of the host fruit fly hemocytes to spread was affected by the interaction between host and parasitoid species (LR χ^2^ = 286.59, df = 2, *p* < 0.001). Overall, *B. dorsalis* had a higher proportion of spread hemocytes compared to *C. cosyra* irrespective of parasitoid species. In both host species, the proportion of spread hemocytes reduced after exposure to parasitoids with the lowest proportion of spread hemocytes recorded in the *D. longicaudata*-parasitized larval groups (7.23% ± 0.21% for *B. dorsalis* and 2.75% ± 0.19% for *C. cosyra*) (Figure 8).

### 3.7 Effects of parasitisation on the viability of *B. dorsalis* and *C. cosyra* hemocytes

**FIGURE 9:**
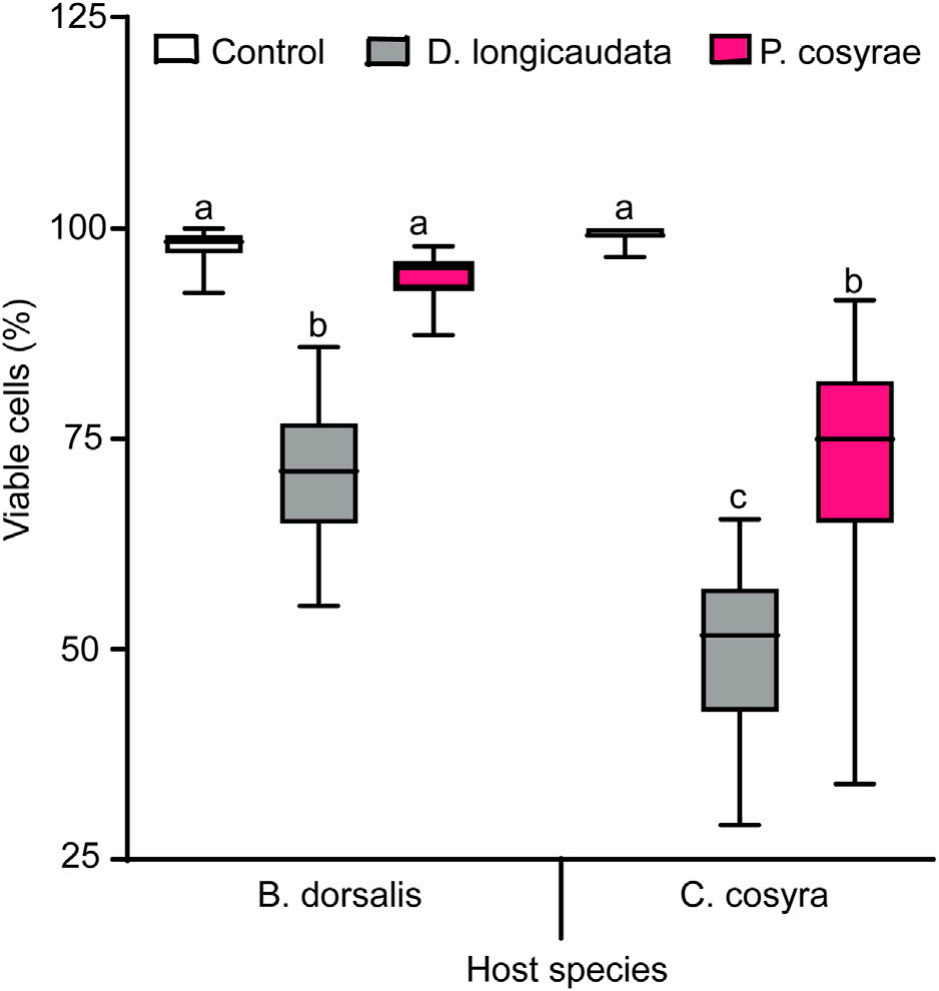
Viable hemocytes of *B. dorsalis* and *C. cosyra* analyzed 36 h after exposure to *D. longicaudata* and *P. cosyra*e: Within each host fruit fly species, boxes with different lower-case letters are statistically different (Tukey tests: *p* ≤ 0.05, *n* = 60). Within each box, horizontal lines denote the median values and the ends of each boxplot whisker represent the minimum and maximum of the data.

The interaction between host and parasitoid species significantly affected hemocyte viability (LR χ^2^ = 51.09, df = 2, *p* < 0.001). In the non-parasitized larval groups, *C. cosyra* had more viable hemocytes (99.35 ± 0.094%) compared to *B. dorsalis* (97.99 ± 0.226%), but the trend was reversed after exposure to parasitoids with *C. cosyra* having lower number of viable hemocytes than *B. dorsalis* regardless of parasitoid species (Figure 9). Particularly, parasitized *C. cosyra* had lower viable hemocyte counts compared to controls regardless of parasitoid species, with the largest reduction (47% relative to the control) recorded in *D. longicaudata*-parasitized larvae. On the other hand, viable hemocyte counts in *B. dorsalis* were only different from that of control when this host was parasitized by *D. longicaudata* (Figure 9).

### 3.8 Effect of host-parasitoid combinations on parasitism success

**FIGURE 10:**
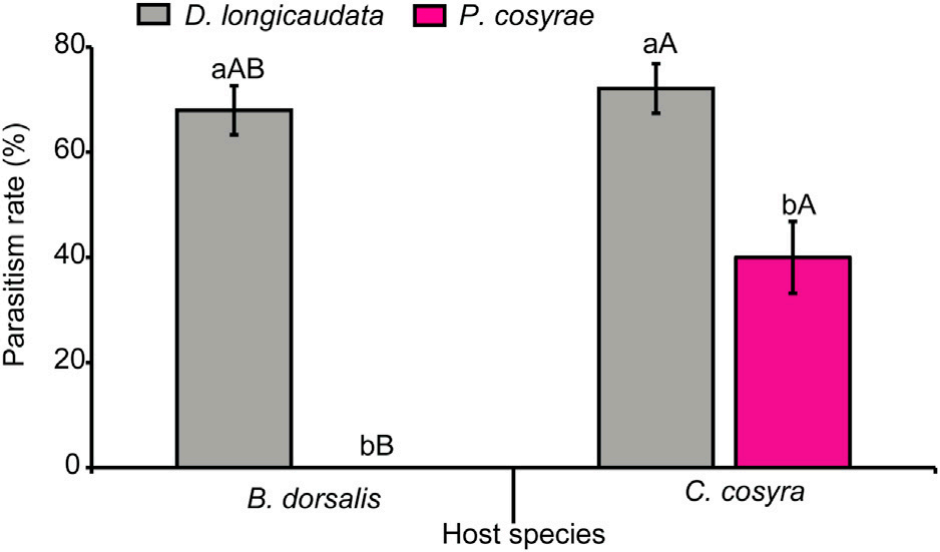
Parasitism rates of *D. longicaudata* and *P. cosyra*e exposed to *B. dorsalis* and *C. cosyra*. Within each host fruit fly species, bars with different letters (lower-case letters denote differences between the different parasitoids exposed to the same host while upper case letters denote differences between the different host species exposed to the same parasitoid species) are statistically different (Tukey tests: *p* ≤ 0.05, *n* = 400). Error bars indicate ±SEM.

Parasitism success was significantly affected by the interaction between host and parasitoid species (LR χ^2^ = 38.50, df = 1, *p* < 0.001). Overall, *D. longicaudata* had higher emergence rates when exposed to both host species (72.13% for *C. cosyra*; 68% for *B. dorsalis*) compared to *P. cosyra*e. Additionally, *C. cosyra* yielded the highest number of parasitoids compared to *B. dorsalis* regardless of parasitoid species. No parasitoids emerged from the *B. dorsalis*-*P. cosyra*e host-parasitoid combination (Figure 10).

**FIGURE 11:**
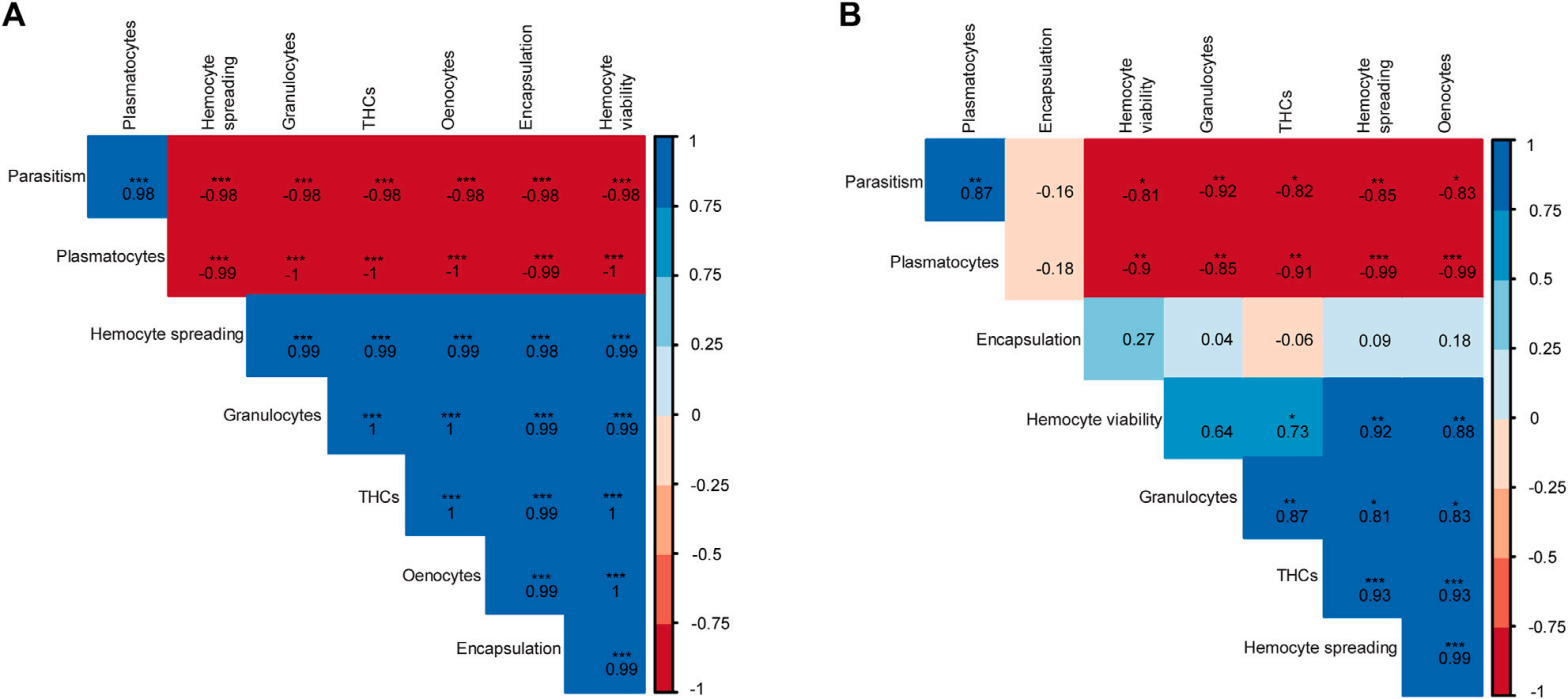
Correlograms showing the relationships between the different host-parasitoid response phenotypes of **(A)** *B. dorsalis* and **(B)** *C. cosyra* exposed to *D. longicaudata* and *P. cosyra*e. Asterisks represent significant correlations (*** for *p* < 0.001, ** for *p* < 0.01, and * for *p* < 0.05). The numbers in each box indicate the Pearson correlation coefficients (r) for the respective pair of parameters compared.

To investigate the correlations between the immune and parasitism emergence phenotypes, a Pearson’s correlation was performed and correlograms were constructed for the respective parameters of each host fruit fly (Figures 11A,B). For *B. dorsalis*, we found that parasitism success was significant but negatively correlated with encapsulation, THCs, granulocyte and oenocyte counts as well as hemocyte spreading and viability indices but was positively correlated with plasmatocyte counts (Figure 11A). On the other hand, encapsulation was positively correlated with THCs, granulocyte counts and hemocyte spreading abilities but negatively correlated with plasmatocyte counts. However, for *C. cosyra*, there was a negative albeit non-significant correlation between encapsulation and parasitism success. However, we recorded significant negative correlations between parasitism success and THCs, hemocyte spreading and viability indices as well as oenocyte, granulocyte and plasmatocyte counts and a significant positive correlation between plasmatocyte counts and parasitoid emergence (Figure 11B).

## 4 Discussion

Both *B. dorsalis* and *C. cosyra* encapsulated the eggs of their parasitic wasps, *D. longicaudata* and *P. cosyra*e but at varying degree. *Bactrocera dorsalis* encapsulated more parasitoid eggs than *C. cosyra*, suggesting that *B. dorsalis* mounts a stronger immune response against both parasitic wasps. Notably, all *P. cosyra*e eggs in *B. dorsalis* larvae were melanotically encapsulated while only up to 37% of *D. longicaudata* eggs were encapsulated in this fruit fly species. These results demonstrate that *B. dorsalis*’ immune system can overcome immune suppression factors produced by *P. cosyra*e but is not strong enough to overcome the host immune-evasive mechanisms employed by *D. longicaudata*. However, contrary to our findings, Mohamed et al. (2008) reported up to 70% encapsulation rates in *B. dorsalis* when exposed to *D. longicaudata*, a value almost two times higher than the one recorded in our study. The distinctly lower encapsulation rates of *D. longicaudata* by *B. dorsalis* recorded in our study could be accounted for by increased transgenerational parasitoid virulence which has improved performance of *D. longicaudata* on its host, *B. dorsalis*. *Diachasmimorpha longicaudata* parasitoids used in the current study were the 176^th^ generation of the initially introduced parasitoids from Hawaii, while those used by Mohamed et al. (2008) were from the 16^th^ generation of the same parasitoid culture. Hence, the continuous rearing of *D*. *longicaudata* on *B. dorsalis* may have contributed to additive transgenerational parasitoid virulence which in turn could explain the reduced host encapsulation rates of *D. longicaudata* by *B*. *dorsalis*. The phenomenon of improved parasitoid virulence has been demonstrated in other parasitoid-host associations (e.g., Carton et al., 1989; Henter and Via, 1995; Jarrett et al., 2022). Nevertheless, the finding that *B. dorsalis* melanotically encapsulated all *P. cosyra*e eggs corroborates previous reports (Ndlela et al., 2020) and further depicts physiological incompatibility between *P. cosyra*e and *B. dorsalis* implying that this parasitoid is unsuitable as a biological control agent of *B. dorsalis*.

Interestingly, *C. cosyra* exhibited a very weak immune response to parasitization by both, *D. longicaudata* and *P. cosyra*e, whereby all eggs (except two) oviposited in this host were free from encapsulation and/or melanisation. These are interesting results since other congeneric tephritids have been shown to mount high encapsulation rates accompanied by melanisation when exposed to parasitoids (Mohamed et al., 2003, 2007, 2008; Suárez et al., 2020). It therefore appears that *C. cosyra* differs from other Ceratitis species in terms of physiological responses against parasitoids and these could likely be due to parasitoid-mediated suppression of factors mediating melanisation or that melanotic encapsulation in *C. cosyra* occurs later than 36hpp. Nevertheless, the poor immune response of *C. cosyra* to *P. cosyra*e in understandable, since the parasitoid shares co-evolutionary history with the pest, unlike *D. longicaudata* that was recently introduced into the African ecosystem.

Encapsulation rates also varied among the two parasitoid species: less *D. longicaudata* eggs were encapsulated relative to *P. cosyra*e eggs. This could be attributed to the presence of viruses in the venom gland of *D. longicaudata*. Viruses including the entomopox virus, DlEPV (Lawrence and Akin, 1990; Khoo and Lawrence, 2002; Lawrence, 2002), a rabdho virus, DlRhv (Lawrence, 1988; Lawrence and Matos, 2005) as well as a rod-shaped virus (Luo and Zeng, 2010) are reported to be associated with this parasitoid. The DlEPV has been shown to invade the host hemocytes where it replicates and exerts cytopathic effects (Lawrence, 1988, 2005). It is, therefore, likely that these viruses played a role in the downregulation of encapsulation and melanisation responses of *D. longicaudata*-parasitized hosts.

Currently, we can only speculate about the mechanisms by which *P. cosyra*e suppresses host immune defenses. However, the venoms of closely related parasitoid species, *Psyttalia concolor* (Szépligeti) and *Psyttalia lonsburyi* (Silvestri) (both Hymenoptera: Braconidae) have been shown to contain leucine-rich repeat proteins (Mathé-Hubert et al., 2016) which is known to interfere with innate immune functioning in host insects (Ng and Xavier, 2011). Due to the low interspecific variation in venom composition of previously described *Psyttalia* species (Mathé-Hubert et al., 2016), it is possible that *P. cosyra*e venom also has the same contents that mediate the observed host immune phenotypes in *P. cosyra*e-parasitized larval groups.

Furthermore, significant reductions in the spreading ability of hemocytes of *B. dorsalis* and *C. cosyra* post-parasitisation by *D. longicaudata* and *P. cosyra*e were recorded. Hemocyte spreading is a prerequisite to the encapsulation response and its inhibition remarkedly reduces host defense mechanisms (Dean et al., 2004). Suppression of hemocyte spreading occurs via the inhibition of the plasmatocyte spreading peptide (Strand and Clark, 1999; Srikanth et al., 2011) or injection of GTPase activating protein P4 (Labrosse et al., 2005) and Rac-GTPase (Williams et al., 2005, 2006), which alter cytoskeletal rearrangement and hemocyte adhesion properties (Carton et al., 2008; Kim and Kim, 2010). We, therefore, postulate that both *D. longicaudata* and *P. cosyra*e injected substances that interfered with their hosts’ hemocyte cytoskeletal rearrangement and adhesion properties, hence inhibiting hemocyte spreading. This has been demonstrated in other parasitized host species (Rizki and Rizki, 1994; Labrosse et al., 2005; Williams et al., 2005, 2006) and those injected with parasitoid venom (Richards and Parkinson, 2000).

In terms of THCs, we found that, *B. dorsalis* had significantly higher THCs which were positively correlated with the encapsulation rates of parasitoid eggs in this host. Indeed, elevated THCs have been reported to be associated with higher encapsulation rates in related Dipterans. For example, Kacsoh and Schlenke (2012) reported higher melanotic encapsulation and stronger wasp resistance phenotypes in *Drosophilla suzukii* (Matsumura) (Diptera: Drosophilidae), which had twice as many circulating hemocyte numbers as its counterpart *Drosophilla melanogaster* (Meigen) (Diptera: Drosophilidae). Similarly, Eslin and Prevost. (1996) and Moreau et al. (2005) showed that drosophilids with higher THCs mounted stronger immune responses, findings which corroborate our hypothesis that hosts with higher hemocyte counts mount stronger immune responses against natural enemies. On the other hand, we recorded remarkable reductions in the number of viable cells among the parasitized larval groups and further observed pyknosis-karyorrhexis-like characteristics in the nuclei of the parasitized larval hemocytes. Although not definitive, these findings are suggestive of parasitoid-induced apoptosis which certainly explains the low THCs in the parasitized larval host groups relative to the controls. In addition, the finding that host larvae exposed to *D. longicaudata* had lower THCs and lower proportions of live hemocytes suggests that this parasitoid induces cytopathic immunosuppressive effects on its hosts’ immunity. Similar cytopathic phenotypes were recorded in the hemocytes of *Anastrepha suspensa* (Loew) (Diptera: Tephritidae) exposed to *D. longicaudata* (Lawrence, 2005). On the other hand, continuous recruitment of circulating hemocytes to form capsules around the parasitoid egg could also account for the lower THCs in the parasitized larval groups of both host species relative to their controls.

Interestingly, we observed significant post-parasitisation changes in the constitutive hemocyte counts of both host insects. Granulocyte numbers of both *C. cosyra* and *B. dorsalis* increased post parasitisation, indicating parasitoid-induced differentiation and release of granulocytes into circulation. However, granulocyte counts recorded in *B. dorsalis* were three-fold as that of *C. cosyra*. Since these cells are central to the encapsulation and melanisation responses, we speculate that they are partly responsible for the higher encapsulation and melanisation phenotypes observed in *B. dorsalis*. Contrary to the increasing granulocyte number, we observed a general decrease in the plasmatocyte counts of both *B. dorsalis* and *C. cosyra* post-parasitisation. The observed trend of increasing granulocytes and decrease in plasmatocyte counts could be as a result of plasmatocyte differentiation into granulocytes. This phenomenon has been demonstrated in other insect taxa (Gupta and Sutherland, 1966; Silva et al., 2002; Richardson et al., 2018). In addition, exposing host insects to parasitic wasps has been associated with selective plasmatocyte-apoptosis (Chiu and Govind, 2002; Wan et al., 2020). We therefore argue that, by inducing targeted apoptosis of plasmatocytes, *D. longicaudata* and *P. cosyra*e reduced the plasmatocyte numbers of their hosts, *B. dorsalis* and *C. cosyra*.

Among the DHCs that we recorded, the roles of spherulocytes and adipocytes in innate immunity are unknown, however, oenocytes have been reported to mediate melanisation by activating the phenoloxidase cascade (Shrestha and Kim, 2008; Strand, 2008). Thus, the observed increment in oenocyte numbers post parasitisation could be a host response aimed at upscaling the phenoloxidase cascade to facilitate melanisation of the parasitoid egg. The correspondingly higher melanisation rates observed in *B. dorsalis* corroborate our argument.

There are two schools of thought regarding the formation of giant multinucleated hemocytes. The first one suggests that these cells are formed due to interference with the cell division process (Hao et al., 2018). The second theory suggests that these hemocytes are formed as a result of the fusion of one or more cells (Markus et al., 2015; Cinege et al., 2020). Since we did not observe any malformed spindles nor incomplete cytokinesis in the multinucleated hemocytes of *B. dorsalis*, our findings are more in line with the second theory which suggests that these cells are formed due to the fusion of several hemocytes. A study by Cinege et al. (2020) showed that GMHs were present in both immune- and non-immune challenged *Zaprionus indianus* Gupta (Diptera: Drosophilidae) and that GMH counts increased post-parasitisation. Notwithstanding, we found that GMHs were only present in parasitized *B. dorsalis* larvae; this suggests that the formation of GMHs is species-specific and only occurs following immune challenge.

The higher GMH counts recorded in *D. longicaudata*-parasitized larval groups relative to those parasitized by *P. cosyra*e could be due to 1) symbiotic virus-induced formation of GMHs via cell-to cell fusion as a mechanism to spread from one cell to another in infected hosts (Albrecht et al., 1996; García-Murria et al., 2019). Since DlEPV, a symbiotic virus associated with *D. longicaudata* invades and replicates in host hemocytes (Lawrence, 2005), it is possible that it induces cell fusion to facilitate its spread among the host hemocytes. 2) formation of GMHs could be a defensive mechanism against parasitoid attack since GMHs actively participate in the encapsulation of parasitoid eggs (Markus et al., 2015).

As predicted, we found that *D. longicaudata* successfully emerged from both host species but *P. cosyra*e adult emergence was only recorded for its sympatric host, *C. cosyra*. These results depict stronger immune evasive mechanisms employed by *D. longicaudata* likely via injecting immune suppressive viruses and further compound the findings by Ndlela et al. (2020) that *B. dorsalis* is an unsuitable host for *P. cosyra*e and that this fruit fly host is a reproductive sink for *P. cosyra*e. The lower emergence rates of *D. longicaudata* when exposed to *B. dorsalis* compared to *C. cosyra* could be attributed to the higher hemocyte counts in *B. dorsalis* that likely neutralize the *D. longicaudata*-venom induced cytopathic effects such that this host can still encapsulate *D. longicaudata* eggs. The positive correlation between THCs and encapsulation rates recorded for this host species corroborates our argument.

We also expected parasitism success and encapsulation rates to be significantly correlated in both *C. cosyra* and *B. dorsalis*. However, while this assumption held true for *B. dorsalis*, there was no significant correlation between encapsulation and parasitism success for *C. cosyra*. Thus, it seems unlikely that melanotic encapsulation is the main defense mechanism employed by *C. cosyra* when attacked by parasitic wasps. It is also possible that the thin hemocyte capsule formed by *C. cosyra* failed to fully develop or degraded, enabling the few encapsulated eggs to survive and develop into adult wasps.

## 5 Conclusion

Taken together, this study demonstrates that host cellular immune responses are key mediators of host-parasitoid dynamics of *B. dorsalis* and *C. cosyra* exposed to the parasitic wasps, *D. longicaudata* and *P. cosyra*e. *Bactrocera dorsalis* exhibited stronger immune defenses which are central to *B. dorsalis*’ resistance against parasitism and likely contribute to its successful invasion and establishment across several ecological zones.

Additionally, that *D. longicaudata* markedly reduced host immune defenses suggests that this parasitoid engages active immune suppression mechanisms to evade host defense mechanisms. This partly explains the high parasitism rates achieved by *D. longicaudata*, making it a formidable control agent for many tephritid fruit fly pests.

In this regard, the introduction and release of *D. longicaudata* in the African eco-system presents a vital addition to the biological control of *C. cosyra* and *B. dorsalis*.

## Data Availability

The datasets presented in this study can be found in online repositories. The names of the repository/repositories and accession number(s) can be found below: https://dmmg.icipe.org/dataportal/dataset/differential-immune-responses-in-new-and-old-fruit-fly-parasitoid-association.

## References

[B1] Albrecht T. Fons M. Boldogh I. Rabson A. S. (1996). “Effects on cells,” in Medical microbiology. Editor Baron S. (Galveston, TX: Galveston (TX): University of Texas Medical Branch at Galveston). Available at: https://www.ncbi.nlm.nih.gov/books/NBK7627/(Accessed February 14, 2022). 21413282

[B2] Altuntaş H. Kiliç A. Y. Uçkan F. Ergin E. (2012). Effects of gibberellic acid on hemocytes of *Galleria mellonella* L. (Lepidoptera: Pyralidae). Environ. Entomol. 41, 688–696. 10.1603/EN11307 22732628

[B3] Binggeli O. Neyen C. Poidevin M. Lemaitre B. (2014). Prophenoloxidase activation is required for survival to microbial infections in Drosophila. PLoS Pathog. 10, e1004067. 10.1371/JOURNAL.PPAT.1004067 24788090 PMC4006879

[B4] Carton Y. Capy P. Nappi A. (1989). Genetic variability of host-parasite relationship traits: Utilization of isofemale lines in a *Drosophila simulans* parasitic wasp. Genet. Sel. Evol. 21, 437. 10.1186/1297-9686-21-4-437

[B5] Carton Y. Poirié M. Nappi A. J. (2008). Insect immune resistance to parasitoids. Insect Sci. 15, 67–87. 10.1111/J.1744-7917.2008.00188.X

[B6] Chiu H. Govind S. (2002). Natural infection of *D. melanogaster* by virulent parasitic wasps induces apoptotic depletion of hematopoietic precursors. Cell Death Differ. 9, 1379–1381. 10.1038/sj.cdd.4401134 12478476

[B7] Cho Y. Cho S. (2019). Hemocyte-hemocyte adhesion by granulocytes is associated with cellular immunity in the cricket, *Gryllus bimaculatus* . Sci. Rep. 9, 18066–18112. 10.1038/s41598-019-54484-5 31792279 PMC6889498

[B8] Cinege G. Lerner Z. Magyar L. B. Soós B. Tóth R. Kristó I. (2020). Cellular immune response involving multinucleated giant hemocytes with two-step genome amplification in the *drosophilid Zaprionus indianus* . J. Innate Immun. 12, 257–272. 10.1159/000502646 31553970 PMC7265743

[B9] Coates C. J. Lim J. Harman K. Rowley A. F. Griffiths D. J. Emery H. (2019). The insect, *Galleria mellonella*, is a compatible model for evaluating the toxicology of okadaic acid. Cell Biol. Toxicol. 35, 219–232. 10.1007/s10565-018-09448-2 30426330 PMC6556153

[B10] Cribari-Neto F. Zeileis A. (2010). Beta regression in R. J. Stat. Softw. 34, 129–150. 10.18637/jss.v034.i02

[B11] Dean P. Potter U. Richards E. H. Edwards J. P. Charnley A. K. Reynolds S. E. (2004). Hyperphagocytic haemocytes in *Manduca sexta* . J. Insect Physiol. 50, 1027–1036. 10.1016/j.jinsphys.2004.09.003 15607505

[B12] Dudzic J. P. Kondo S. Ueda R. Bergman C. M. Lemaitre B. (2015). *Drosophila* innate immunity: Regional and functional specialization of prophenoloxidases. BMC Biol. 13, 81. 10.1186/S12915-015-0193-6 26437768 PMC4595066

[B13] Ekesi S. Nderitu P. W. Rwomushana I. (2006). Field infestation, life history and demographic parameters of the fruit fly *Bactrocera invadens* (Diptera: Tephritidae) in Africa. Bull. Entomol. Res. 96, 379–386. 10.1079/BER2006442 16923206

[B14] Eslin P. Doury G. G. (2006). The fly *Drosophila subobscura*: A natural case of innate immunity deficiency. Dev. Comp. Immunol. 30, 977–983. 10.1016/j.dci.2006.02.007 16620975

[B15] Eslin P. Prévost G. (1998). Hemocyte load and immune resistance to *Asobara tabida* are correlated in species of the *Drosophila melanogaster* subgroup. J. Insect Physiol. 44, 807–816. 10.1016/S0022-1910(98)00013-4 12769876

[B16] Eslin P. Prévost G. (2000). Racing against host’s immunity defenses: A likely strategy for passive evasion of encapsulation in *Asobara tabida* parasitoids. J. Insect Physiol. 46, 1161–1167. 10.1016/S0022-1910(99)00227-9 10818243

[B17] Eslin P. Prevost G. (1996). Variation in *Drosophila* concentration of haemocytes associated with different ability to encapsulate *Asobara tabida* larval parasitoid. J. Insect Physiol. 42, 549–555. 10.1016/0022-1910(95)00134-4

[B18] García-Murria M. J. Expósito-Domínguez N. Duart G. Mingarro I. Martinez-Gil L. (2019). A bimolecular multicellular complementation system for the detection of syncytium formation: A new methodology for the identification of nipah virus entry inhibitors. Viruses 11, E229. 10.3390/v11030229 30866435 PMC6466393

[B19] Gupta A. P. (1979). “Hemocyte types: Their structures, synonymies, interrelationships, and taxonomic significance,” in Insect hemocytes: Development, forms, functions and techniques. Editor Gupta A. (Cambridge, United Kingdom: Cambridge University Press), 85–128. 10.1017/cbo9780511759987.005

[B20] Gupta A. P. Sutherland D. J. (1966). *In vitro* transformations of the insect plasmatocyte in some insects. J. Insect Physiol. 12, 1369–1375. 10.1016/0022-1910(66)90151-X

[B21] Gwokyalya R. Altuntaş H. (2019). Boric acid-induced immunotoxicity and genotoxicity in model insect *Galleria mellonella* L. (Lepidoptera: Pyralidae). Arch. Insect Biochem. Physiol. 101, e21588. 10.1002/arch.21588 31180585

[B22] Hao Y. Yu S. Luo F. Jin L. H. (2018). Jumu is required for circulating hemocyte differentiation and phagocytosis in *Drosophila* . Cell Commun. Signal. 16 (1), 95–20. 10.1186/S12964-018-0305-3 30518379 PMC6280549

[B23] Harbi A. De Pedro L. Ferrara F. A. A. Tormos J. Chermiti B. Beitia F. (2019). *Diachasmimorpha longicaudata* parasitism response to medfly host fruit and fruit infestation age. Insects 10, E211–E212. 10.3390/insects10070211 31323827 PMC6681355

[B24] Henter H. J. Via S. (1995). The potential for coevolution in a host-parasitoid system. I. Genetic variations within an aphid population in susceptibility to a parasitic wasp. Evolution 49, 427–438. 10.1111/j.1558-5646.1995.tb02275.x 28565087

[B25] Jarrett B. J. M. Linder S. Fanning P. D. Isaacs R. Szűcs M. (2022). Experimental adaptation of native parasitoids to the invasive insect pest, *Drosophila suzukii* . Biol. Control 167, 104843. 10.1016/j.biocontrol.2022.104843

[B26] Kacsoh B. Z. Schlenke T. A. (2012). High hemocyte load is associated with increased resistance against parasitoids in *Drosophila suzukii*, a relative of *D. melanogaster* . PLoS One 7, e34721. 10.1371/journal.pone.0034721 22529929 PMC3328493

[B27] Khoo C. C. H. Lawrence P. O. (2002). Hagen’s glands of the parasitic wasp *Diachasmimorpha longicaudata* (Hymenoptera: Braconidae): Ultrastructure and the detection of entomopoxvirus and parasitism-specific proteins. Arthropod Struct. Dev. 31, 121–130. 10.1016/S1467-8039(02)00021-X 18088975

[B28] Kim G. S. Kim Y. (2010). Up-regulation of circulating hemocyte population in response to bacterial challenge is mediated by octopamine and 5-hydroxytryptamine via Rac1 signal in *Spodoptera exigua* . J. Insect Physiol. 56, 559–566. 10.1016/j.jinsphys.2009.11.022 19961854

[B29] Labrosse C. Stasiak K. Lesobre J. Grangeia A. Huguet E. Drezen J. M. (2005). A RhoGAP protein as a main immune suppressive factor in the *Leptopilina boulardi* (Hymenoptera, Figitidae)-*Drosophila melanogaster* interaction. Insect biochem. Mol. Biol. 35, 93–103. 10.1016/j.ibmb.2004.10.004 15681220

[B30] Lawrence P. O. Akin D. (1990). Virus-like particles from the poison glands of the parasitic wasp Biosteres longicaudatus (Hymenoptera: Braconidae). Can. J. Zool. 68, 539–546. 10.1139/z90-079

[B31] Lawrence P. O. (1988). Ecdysteroid titres and integument changes in superparasitized puparia of *Anastrepha suspensa* (Diptera: Tephritidae). J. Insect Physiol. 34, 603–608. 10.1016/0022-1910(88)90066-2

[B32] Lawrence P. O. Matos L. F. (2005). Transmission of the *Diachasmimorpha longicaudata* rhabdovirus (DlRhV) to wasp offspring: An ultrastructural analysis. J. Insect Physiol. 51, 235–241. 10.1016/j.jinsphys.2005.01.002 15749107

[B33] Lawrence P. O. (2005). Morphogenesis and cytopathic effects of the *Diachasmimorpha longicaudata* entomopoxvirus in host haemocytes. J. Insect Physiol. 51, 221–233. 10.1016/j.jinsphys.2004.12.003 15749106

[B34] Lawrence P. O. (2002). Purification and partial characterization of an entomopoxvirus (DlEPV) from a parasitic wasp of tephritid fruit flies. J. Insect Sci. 2, 10–12. 10.1093/jis/2.1.10 15455044 PMC355910

[B35] Leblanc L. Vargas R. Putoa R. (2013). From eradication to containment: Invasion of French Polynesia by *Bactrocera dorsalis* (hendel) (Diptera: Tephritidae) and releases of two natural enemies: A 17-year case study. in Proceedings of the Hawaiian entomological society, Hawaii, December, 31–43.

[B36] Luo L. Zeng L. (2010). A new rod-shaped virus from parasitic wasp *Diachasmimorpha longicaudata* (Hymenoptera: Braconidae). J. Invertebr. Pathol. 103, 165–169. 10.1016/j.jip.2009.08.008 19682456

[B37] Markus R. Lerner Z. Honti V. Csordas G. Zsamboki J. Cinege G. (2015). Multinucleated giant hemocytes are effector cells in cell-mediated immune responses of *Drosophila* . J. Innate Immun. 7, 340–353. 10.1159/000369618 25659341 PMC6738834

[B38] Mathé-Hubert H. Colinet D. Deleury E. Belghazi M. Ravallec M. Poulain J. (2016). Comparative venomics of *Psyttalia lounsburyi* and *P. concolor*, two olive fruit fly parasitoids: A hypothetical role for a GH1 β-glucosidase. Sci. Rep. 6, 35873. 10.1038/srep35873 27779241 PMC5078806

[B39] Mohamed S. A. Ekesi S. Hanna R. (2008). Evaluation of the impact of *Diachasmimorpha longicaudata* on *Bactrocera invadens* and five African fruit fly species. J. Appl. Entomol. 132, 789–797. 10.1111/J.1439-0418.2008.01350.X

[B40] Mohamed S. A. Overholt W. A. Lux S. A. Wharton R. A. Eltoum E. M. (2007). Acceptability and suitability of six fruit fly species (Diptera: Tephritidae) for Kenyan strains of *Psyttalia concolor* (Hymenoptera: Braconidae). Biocontrol Sci. Technol. 17, 247–259. 10.1080/09583150701211418

[B41] Mohamed S. A. Overholt W. A. Wharton R. A. Lux S. A. Eltoum E. M. (2003). Host specificity of *Psyttalia cosyrae* (Hymenoptera: Braconidae) and the effect of different host species on parasitoid fitness. Biol. Control 28, 155–163. 10.1016/S1049-9644(03)00099-9

[B42] Mohamed S. A. Ramadan M. M. Ekesi S. (2016). “In and out of Africa: Parasitoids used for biological control of fruit flies,” in Fruit fly research and development in Africa - towards a sustainable management strategy to improve horticulture. Editor Ekesi S.. (Salmon Tower Building: Springer International Publishing), 325–368. 10.1007/978-3-319-43226-7_16

[B43] Monconduit H. Prevost G. Fourdrain Y. (1994). Avoidance of encapsulation by *Asobara tabida*, a larval parasitoid of *Drosophila* species. Can. J. Zool. 74, 2193–2198. 10.1139/z96-248

[B44] Moreau S. J. M. Guillot S. Populaire C. Doury G. Prévost G. Eslin P. (2005). Conversely to its sibling *Drosophila melanogaster, D. simulans* overcomes the immunosuppressive effects of the parasitoid *Asobara citri* . Dev. Comp. Immunol. 29, 205–209. 10.1016/j.dci.2004.07.002 15572069

[B45] Mwatawala M. W. De Meyer M. Makundi R. H. Maerere A. P. (2006). Biodiversity of fruit flies (Diptera, Tephritidae) in orchards in different agro-ecological zones of the Morogoro region, Tanzania. Fruits 61, 321–332. 10.1051/fruits:2006031

[B46] Nappi A. Poirié M. Carton Y. (2009). The role of melanization and cytotoxic by-products in the cellular immune responses of *Drosophila* against parasitic wasps. Adv. Parasitol. 70, 99–121. 10.1016/S0065-308X(09)70004-1 19773068

[B47] Ndlela S. Mohamed S. A. Azrag A. G. A. Ndegwa P. N. Ong’amo G. O. Ekesi S. (2020). Interactions between two parasitoids of tephritidae: *Diachasmimorpha longicaudata* (ashmead) and *Psyttalia cosyrae* (wilkinson) (Hymenoptera: Braconidae), under laboratory conditions. Insects 11, 6711–E716. 10.3390/insects11100671 PMC7599697 33023254

[B48] Ng A. Xavier R. J. (2011). Leucine-rich repeat (LRR) proteins: Integrators of pattern recognition and signaling in immunity. Autophagy 7, 1082–1084. 10.4161/auto.7.9.16464 21606681 PMC3901792

[B49] Ovruski S. M. Aluja M. Sivinski J. Wharton R. A. (2000). Hymenopteran parasitoids on fruit-infesting Tephritidae (Diptera) in Latin America and the southern United States: Diversity, distribution, taxonomic status and their use in fruit fly biological control. Integr. Pest Manag. Rev. 5, 81–107. 10.1023/A:1009652431251

[B50] Pech L. L. Strand M. R. (1996). Granular cells are required for encapsulation of foreign targets by insect haemocytes. J. Cell Sci. 109, 2053–2060. 10.1242/jcs.109.8.2053 8856501

[B51] Richards E. H. Parkinson N. M. (2000). Venom from the endoparasitic wasp Pimpla hypochondriaca adversely affects the morphology, viability, and immune function of hemocytes from larvae of the tomato moth, *Lacanobia oleracea* . J. Invertebr. Pathol. 76, 33–42. 10.1006/jipa.2000.4948 10963401

[B52] Richardson R. T. Ballinger M. N. Qian F. Christman J. W. Johnson R. M. (2018). Morphological and functional characterization of honey bee, *Apis mellifera*, hemocyte cell communities. Apidologie 49, 397–410. 10.1007/s13592-018-0566-2

[B53] Rizki T. M. Rizki R. M. (1994). Parasitoid-induced cellular immune deficiency in *Drosophila* . Ann. N. Y. Acad. Sci. 712, 178–194. 10.1111/j.1749-6632.1994.tb33572.x 7910721

[B54] Sá L. P. Alvarenga C. D. dos Santos Z. C. Souza M. das D. da C. da Cruz C. G. (2018). Parasitism of *Diachasmimorpha longicaudata* (Ashmead) on two fruit fly species. Arq. Inst. Biol. 85, 1–5. 10.1590/1808-1657000172017

[B55] Schmid-Hempel P. (2009). Immune defence, parasite evasion strategies and their relevance for “macroscopic phenomena” such as virulence. Philos. Trans. R. Soc. Lond. B Biol. Sci. 364, 85–98. 10.1098/rstb.2008.0157 18930879 PMC2666695

[B56] Schmidt O. Theopold U. Strand M. (2001). Innate immunity and its evasion and suppression by hymenopteran endoparasitoids. Bioessays. 23, 344–351. 10.1002/bies.1049 11268040

[B57] Segura D. F. Viscarret M. M. Ovruski S. M. Cladera J. L. (2012). Response of the fruit fly parasitoid *Diachasmimorpha longicaudata* to host and host-habitat volatile cues. Entomol. Exp. Appl. 143, 164–176. 10.1111/j.1570-7458.2012.01246.x

[B58] Shrestha S. Kim Y. (2008). Eicosanoids mediate prophenoloxidase release from oenocytoids in the beet armyworm *Spodoptera exigua* . Insect biochem. Mol. Biol. 38, 99–112. 10.1016/j.ibmb.2007.09.013 18070669

[B59] Silva J. E. B. Boleli I. C. Simões Z. L. P. (2002). Hemocyte types and total and differential counts in unparasitized and parasitized *Anastrepha obliqua* (Diptera, Tephritidae) larvae. Braz. J. Biol. 62, 689–699. 10.1590/S1519-69842002000400017 12659019

[B60] Srikanth K. Park J. Stanley D. W. Kim Y. (2011). Plasmatocyte-spreading peptide influences hemocyte behavior via eicosanoids. Arch. Insect Biochem. Physiol. 78, 145–160. 10.1002/ARCH.20450 22006534

[B61] Strand M. R. Clark K. D. (1999). Plasmatocyte spreading peptide induces spreading of plasmatocytes but represses spreading of granulocytes. Arch. Insect Biochem. Physiol. 42, 213–223. 10.1002/(SICI)1520-6327(199911)42:3<213::AID-ARCH5>3.0.CO;2-4 10536049

[B62] Strand M. R. (2008). The insect cellular immune response. Insect Sci. 15, 1–14. 10.1111/j.1744-7917.2008.00183.x

[B63] Suárez L. Buonocore Biancheri M. J. Sánchez G. Cancino J. Murúa F. Bilbao M. (2020). Radiation on medfly larvae of tsl vienna-8 genetic sexing strain displays reduced parasitoid encapsulation in mass-reared *Diachasmimorpha longicaudata* (Hymenoptera: Braconidae). J. Econ. Entomol. 113, 1134–1144. 10.1093/jee/toaa062 32307531

[B64] Trainor J. E. Kr P. Mortimer N. T. (2021). Immune cell production is targeted by parasitoid wasp virulence in a *Drosophila*–parasitoid wasp interaction. Pathogens 10, 49. 10.3390/PATHOGENS10010049 33429864 PMC7826891

[B65] Vargas R. Leblanc L. Harris E. J. Manoukis N. C. (2012). Regional suppression of *Bactrocera* fruit flies (Diptera: Tephritidae) in the Pacific through biological control and prospects for future introductions into other areas of the world. Insects 3, 727–742. 10.3390/insects3030727 26466626 PMC4553587

[B66] Vargas R. Stark J. D. Uchida G. K. Purcell M. (1993). Opiine parasitoids (Hymenoptera: Braconidae) of oriental fruit fly (Diptera: Tephritidae) on kauai island, Hawaii: Islandwide relative abundance and parasitism rates in wild and orchard guava habitats. Environ. Entomol. 22, 246–253. 10.1093/ee/22.1.246

[B67] Wan B. Yang L. Zhang J. Qiu L. Fang Q. Yao H. (2020). The venom of the ectoparasitoid wasp *Pachycrepoideus vindemiae* (Hymenoptera: Pteromalidae) induces apoptosis of *Drosophila melanogaster* Hemocytes. Insects 11, 363. 10.3390/INSECTS11060363 PMC7349765 32545289

[B68] Wang X. G. Messing R. H. (2004). Potential interactions between pupal and egg- or larval-pupal parasitoids of tephritid fruit flies. Environ. Entomol. 33, 1313–1320. 10.1603/0046-225X-33.5.1313

[B69] Williams M. J. Ando I. Hultmark D. (2005). *Drosophila melanogaster* Rac2 is necessary for a proper cellular immune response. Genes Cells. 10, 813–823. 10.1111/J.1365-2443.2005.00883.X 16098145

[B70] Williams M. J. Wiklund M. L. Wikman S. Hultmark D. (2006). Rac1 signalling in the *Drosophila* larval cellular immune response. J. Cell Sci. 119, 2015–2024. 10.1242/jcs.02920 16621891

